# Supplementary material for: Disease misclassification in electronic healthcare database studies: Deriving validity indices—A contribution from the ADVANCE project
Source: PLoS One. 2020 Apr 22;15(4):e0231333. doi: 10.1371/journal.pone.0231333 (PMC7176121; doi:10.1371/journal.pone.0231333)
Supplement: S1 Table — (DOCX) [file pone.0231333.s001.docx]

**Table S.1:** Constraints on the input parameters ensuring that the derived parameters belong to the interval [0,1].

| **Known** | | **Constraints** | | |
| --- | --- | --- | --- | --- |
| 1. | $\Pi$, P, SE | $1-P>\Pi\left( 1-SE \right)$ | $P>SE \Pi$ |  |
| 2. | $\Pi, P, SP$ | $P>(1-\Pi)\left( 1-SP \right)$ | $P<1-SP\left( 1-\Pi\right)$ |  |
| 3. | $\Pi, P, PPV$ | $\Pi>P PPV$ | $\Pi<1-\left( 1- PPV \right)P$ |  |
| 4. | $\Pi, P, NPV$ | $\Pi>(1-NPV)\left( 1-P \right)$ | $\Pi<1-NPV\left( 1-P \right)$ |  |
| 5. | $P, SE, SP$ | $\frac{P+SP-1}{SE+SP-1}>0$ | $\frac{P+SP+1}{SE+SP-1}>0$ | $\frac{\mathrm{SE}\left( P+SP \right)-P}{SE+SP-1}<1$ |
| 6. | $P,SE,PPV$ | $P \left( \mathrm{PPV}\text{(1-SE)/SE}+1 \right)$<1 |  |  |
| 7. | $P,SE,NPV$ | $\left( 1-P \right)\frac{1-SE NPV}{1-SE}<1$ |  |  |
| 8. | $P,SP,PPV$ | $P+SP\frac{P(1-PPV)}{1-SP}<1$ |  |  |
| 9. | $P,SP,NPV$ | $P SP>\left( 1-SP \right)\left( 1-P \right)\mathrm{NPV}$ |  |  |
| 10. | $P,PPV,NPV$ |  |  |  |
