# Supplementary material for: Disease misclassification in electronic healthcare database studies: Deriving validity indices—A contribution from the ADVANCE project
Source: PLoS One. 2020 Apr 22;15(4):e0231333. doi: 10.1371/journal.pone.0231333 (PMC7176121; doi:10.1371/journal.pone.0231333)
Supplement: S2 Table — (DOCX) [file pone.0231333.s002.docx]

**Table S.2:** Parameter constraints corresponding to a case-finding algorithm that performs better than chance.

| **Constraints** | |
| --- | --- |
| $P>\Pi\times SE$ | $P<\mathrm{SE}$ |
| $\Pi\times\left( 1-SE \right)<1-P$ | $\mathrm{SP}>(1-P)$ |
| $(1-\Pi)\times SP<1-P$ | $\Pi<\mathrm{PPV}$ |
| $(1-\Pi)\times(1-SP)<P$ | $1-NPV<\Pi$ |
